# Effectiveness and safety of adjunct interventions for external cephalic version in breech presentation: a systematic review and meta-analysis

**DOI:** 10.61622/rbgo/2026rbgo40

**Published:** 2026-10-20

**Authors:** Allanis Cristina Bortoli, Vitor Costa Rozan Fortunato

**Affiliations:** 1 Pontifícia Universidade Católica de Campinas Campinas SP Brazil Pontifícia Universidade Católica de Campinas, Campinas, SP, Brazil.

**Keywords:** Version, fetal, Breech presentation, Cesarean delivery, Tocolytic agents, Anesthesia spinal

## Abstract

**Objective:**

To evaluate the efficacy and safety of adjunct and implementation-related strategies associated with external cephalic version (ECV) in pregnant women with breech presentation.

**Methods:**

Randomized controlled trials comparing adjunct or protocol-based strategies related to ECV (including pharmacological tocolysis, neuraxial anesthesia or analgesia, systemic analgesia, or timing/procedural approaches) with standard care, placebo, or expectant management were included.

**Search strategy:**

MEDLINE (PubMed), Embase, Scopus, Cochrane CENTRAL, and LILACS were searched from inception to February 2026.

**Data selection and extraction:**

Data extraction and risk of bias assessment using the Cochrane RoB 2 tool were performed independently by two reviewers. Dichotomous outcomes were pooled using risk ratios (RR) with 95% confidence intervals (CI) under random-effects models.

**Results:**

Ten randomized controlled trials comprising 2,745 randomized women were included; 2,722 contributed complete outcome data to the primary meta-analysis. Adjunct and implementation-related strategies were associated with higher ECV success rates (RR 1.53; 95% CI 1.15–2.04; I² = 87%). Pharmacological tocolysis demonstrated the largest effect (RR 2.39; 95% CI 1.39–4.11), followed by neuraxial anesthesia (RR 1.75; 95% CI 1.07–2.85) and systemic analgesia (RR 1.39; 95% CI 1.15–1.69). Protocol-based strategies did not significantly improve success rates. No statistically significant reduction in cesarean delivery was observed (RR 0.88; 95% CI 0.76–1.02). Serious maternal and neonatal complications were uncommon.

**Conclusions:**

Adjunct and implementation-related strategies, particularly pharmacological tocolysis and neuraxial anesthesia, increase the likelihood of successful ECV. Although the effect on cesarean delivery remains inconclusive, ECV appears safe when performed in appropriate clinical settings.

## Introduction

Breech presentation at term remains a common obstetric condition and is strongly associated with higher rates of cesarean delivery and related maternal morbidity. External cephalic version (ECV) is recommended as an effective strategy to reduce noncephalic birth and increase the likelihood of vaginal delivery, and professional guidelines support its routine offer in eligible pregnancies.^(1)^ Despite its recognized benefits, ECV success is variable and depends on several maternal, fetal, and procedural factors.

To improve outcomes, multiple adjunct strategies have been proposed, including pharmacological tocolysis, neuraxial anesthesia or analgesia, and modifications in the timing or conduct of the maneuver. Previous systematic reviews suggest that uterine relaxation and maternal comfort may enhance procedural success; however, current evidence remains fragmented regarding the true impact of these adjuncts on clinically relevant outcomes such as cephalic presentation at birth, cesarean delivery rates, and maternal or neonatal safety.^(2,3)^

To address this lack of consensus, we conducted a systematic review and meta-analysis to evaluate the efficacy and safety of adjunct and implementation-related strategies associated with external cephalic version in pregnant women with breech presentation.

Specifically, we aimed to compare external cephalic version success rates, cephalic presentation at birth, cesarean delivery rates, and maternal and neonatal complications, and to assess the certainty of the evidence using the GRADE approach.

## Methods

This systematic review and meta-analysis was conducted in accordance with the Preferred Reporting Items for Systematic Reviews and Meta-Analyses (PRISMA 2020) statement and the Cochrane Handbook for Systematic Reviews of Interventions. The protocol was prospectively registered in the PROSPERO database (CRD420261291574) before data extraction and analysis.

Studies were selected according to the PICO framework.

Population: pregnant women with singleton pregnancies in breech presentation eligible for external cephalic version.

Intervention: Adjunct strategies used during external cephalic version (including pharmacological tocolysis, neuraxial anesthesia or analgesia, systemic analgesia) as well as procedural or protocol-based strategies related to the offering or implementation of ECV (e.g., timing strategies or ECV attempt versus expectant management).

Comparator: placebo, standard care, expectant management/no ECV, or alternative adjunct techniques.

Outcomes: external cephalic version success, cephalic presentation at birth, cesarean delivery, and maternal or neonatal complications.

Study design: randomized controlled trials. Observational studies, case reports, reviews, editorials, conference abstracts without full data, and non-comparative studies were excluded.

### Data source and search strategy

A comprehensive search was conducted in MEDLINE (PubMed), Embase, Scopus, Cochrane CENTRAL, and LILACS from database inception to the final search date (February 2026), without language or publication date restrictions. Controlled vocabulary terms (MeSH, Emtree, DeCS) and free-text keywords related to external cephalic version, breech presentation, tocolysis, anesthesia, and delivery outcomes were combined. Grey literature searches were performed in ClinicalTrials.gov and the WHO International Clinical Trials Registry Platform. Reference lists of included studies were manually screened. The full electronic search strategies for all databases are provided in Supplementary Material chart S1.

### Selection and data extraction process

All records were imported into Rayyan (Qatar Computing Research Institute) for duplicate removal and screening. Two reviewers independently screened titles, abstracts, and full texts. Disagreements were resolved by consensus. The selection process was documented using a PRISMA flow diagram.

Two reviewers independently extracted data using a standardized electronic data extraction form. Extracted information included study characteristics, participant demographics, intervention and comparator details, outcome data, and the number of events per group. Extracted data were cross-checked for accuracy, and disagreements were resolved by consensus. When necessary, study authors were contacted for clarification.

### Risk of bias

Risk of bias was independently evaluated using the Cochrane Risk of Bias 2 (RoB 2) tool across five domains: randomization process, deviations from intended interventions, missing outcome data, measurement of outcomes, and selection of reported results. Disagreements were resolved by consensus.

### Statistical analysis

Dichotomous outcomes were pooled using risk ratios (RR) with 95% confidence intervals. Random-effects meta-analysis (Mantel–Haenszel method) was applied to account for between-study heterogeneity. Statistical heterogeneity was assessed using Cochran's Q test and the I² statistic. Subgroup analyses were prespecified according to intervention category. All analyses were performed using Review Manager (RevMan), version 5.4.

### Certainty of evidence

The certainty of evidence for each outcome was assessed using the GRADE framework, considering risk of bias, inconsistency, indirectness, imprecision, and publication bias. Evidence was classified as high, moderate, low, or very low certainty.

As this study analyzed previously published data, institutional review board approval was not required.

## Results

### Study selection

The study selection process is illustrated in figure 1.

**Figure 1:**
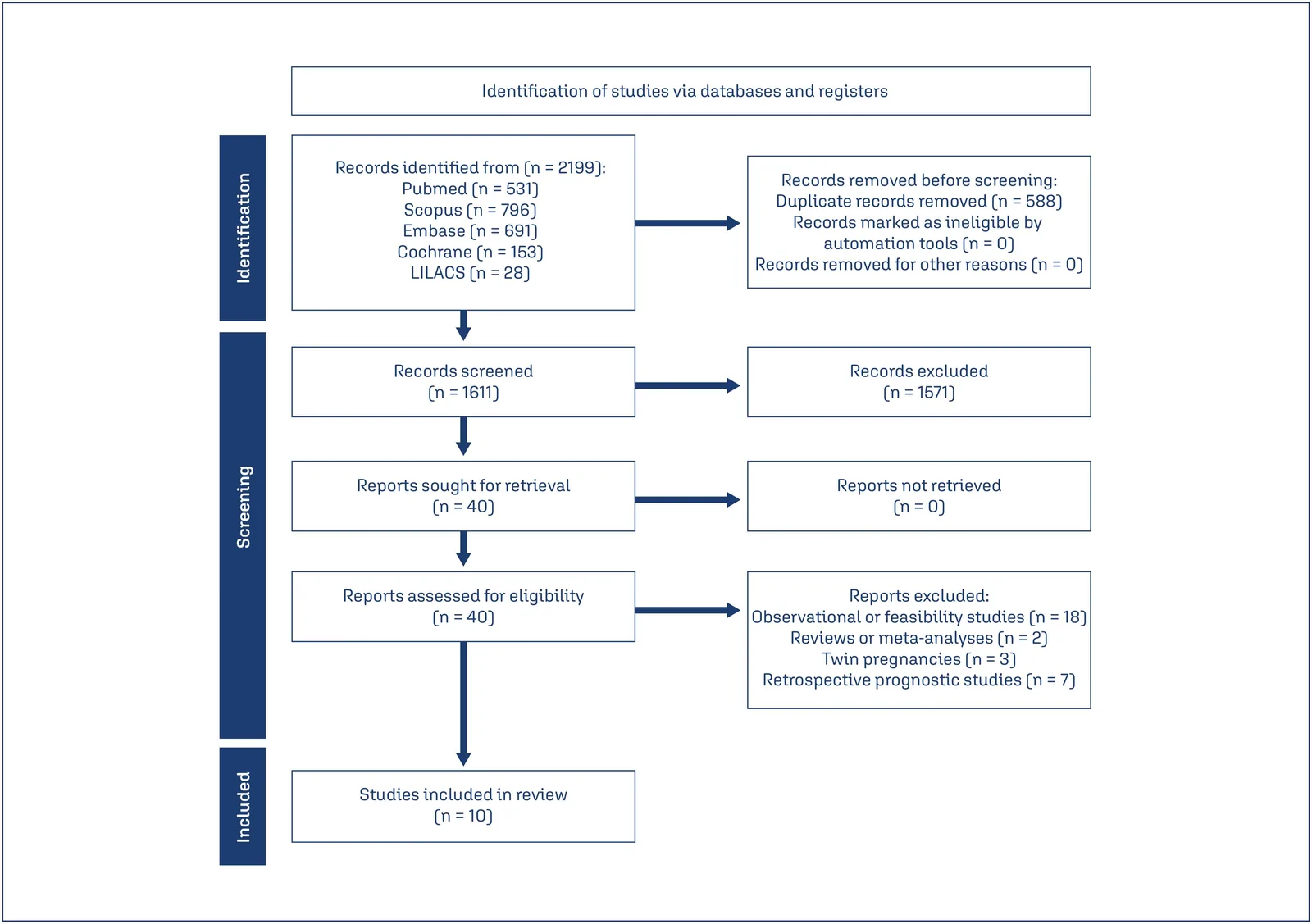
Study selection process.

### Study characteristics

Ten randomized controlled trials met the eligibility criteria and were included in both the qualitative and quantitative synthesis. These studies were published between 1983 and 2022 and collectively randomized 2,745 women. Of these, 2,722 participants had complete outcome data available and were included in the primary meta-analysis. The trials were conducted in South Africa, Malaysia, the United Kingdom, Israel, the United States, Hong Kong, China, and the Netherlands. Detailed study characteristics are summarized in chart 1.

**Chart 1:** Characteristics of the included randomized controlled trials

Study	Country/Setting	Design	Participants randomized (n)	Intervention	Comparator	Primary outcome(s)	Intervention category
Hofmeyr (1983)^(4)^	South Africa	RCT	60	External cephalic version attempt	Expectant management/ no ECV	ECV success	Implementation/protocol strategy
Nor Azlin et al. (2005)^(5)^	Malaysia	RCT	60	Ritodrine tocolysis	Placebo	ECV success	Pharmacological tocolysis
Impey and Pandit (2005)^(6)^	United Kingdom	RCT	124	Intravenous ritodrine tocolysis (repeat ECV)	Placebo	ECV success	Pharmacological tocolysis
Weiniger et al. (2007)^(7)^	Israel	RCT	70	Neuraxial (spinal) anesthesia	Standard care / no anesthesia	ECV success	Neuraxial anesthesia
Sullivan et al. (2009)^(8)^	United States	RCT	96	Combined spinal–epidural anesthesia (CSE)	Systemic opioid analgesia (IV remifentanil)	ECV success	Neuraxial anesthesia
Hutton et al. (2011)^(9)^	Multicenter international	RCT	1541	Early ECV	Delayed/term ECV	Non-cephalic presentation at birth; cesarean delivery	Protocol / timing strategy
Khaw et al. (2015)^(10)^	Hong Kong	RCT	126	Neuraxial anesthesia or remifentanil analgesia	Standard care	ECV success	Analgesia/anesthesia
Liu and Xue (2016)^(11)^	China	RCT	152	IV remifentanil analgesia	Placebo	ECV success	Systemic analgesia
Wang et al. (2017)^(12)^	China	RCT	144	IV remifentanil analgesia	Placebo	ECV success	Systemic analgesia
de Wolff et al. (2022)^(13)^	Netherlands	RCT	372	Rebozo maneuver	Standard care	Cephalic presentation at birth; cesarean delivery	Non-pharmacological strategy

### Intervention categories

Adjunct strategies evaluated across studies were grouped a priori into four categories, including implementation or protocol-based strategies:

- Pharmacological tocolysis (n = 2)^(5,6)^
- Neuraxial anesthesia or analgesia (n = 3)^(7,8,10)^
- Systemic analgesia (n = 3)^(10-12)^
- Protocol or non-pharmacological strategies (n = 3)^(4,9,13)^

The study by Khaw et al. (2015)^(10)^ contributed data to both the neuraxial and systemic analgesia subgroups.

### Risk of bias assessment

Risk of bias was assessed using the Cochrane Risk of Bias 2 (RoB 2) tool across five domains. All studies were classified as low risk for missing outcome data (D3), measurement of the outcome (D4), and selection of the reported result (D5). Nine studies were rated as low risk for the randomization process (D1), and one study was judged as having some concerns. Seven studies were judged as having some concerns in deviations from intended interventions (D2). No study was classified as high risk of bias in any domain or in the overall assessment as shown figures 2 and 3.

**Figure 2:**
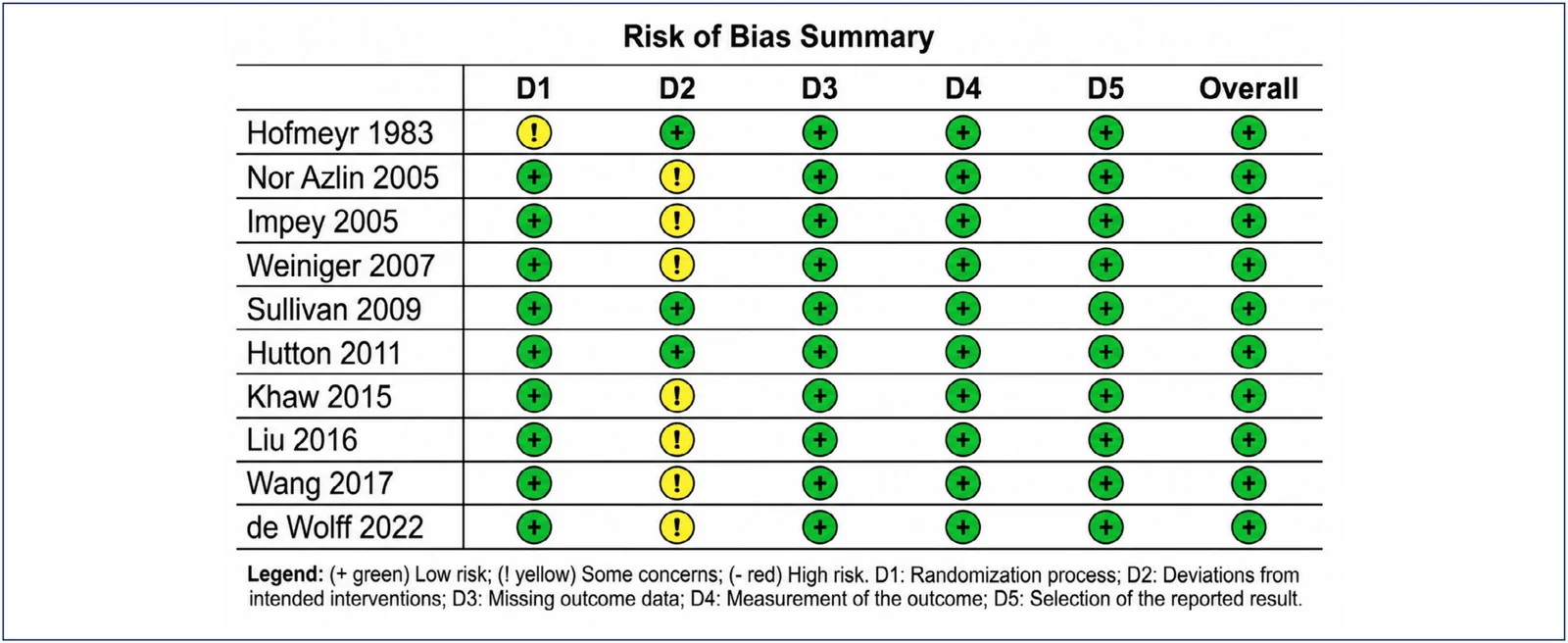
Risk of bias summary across RoB domains for the included studies

**Figure 3:**
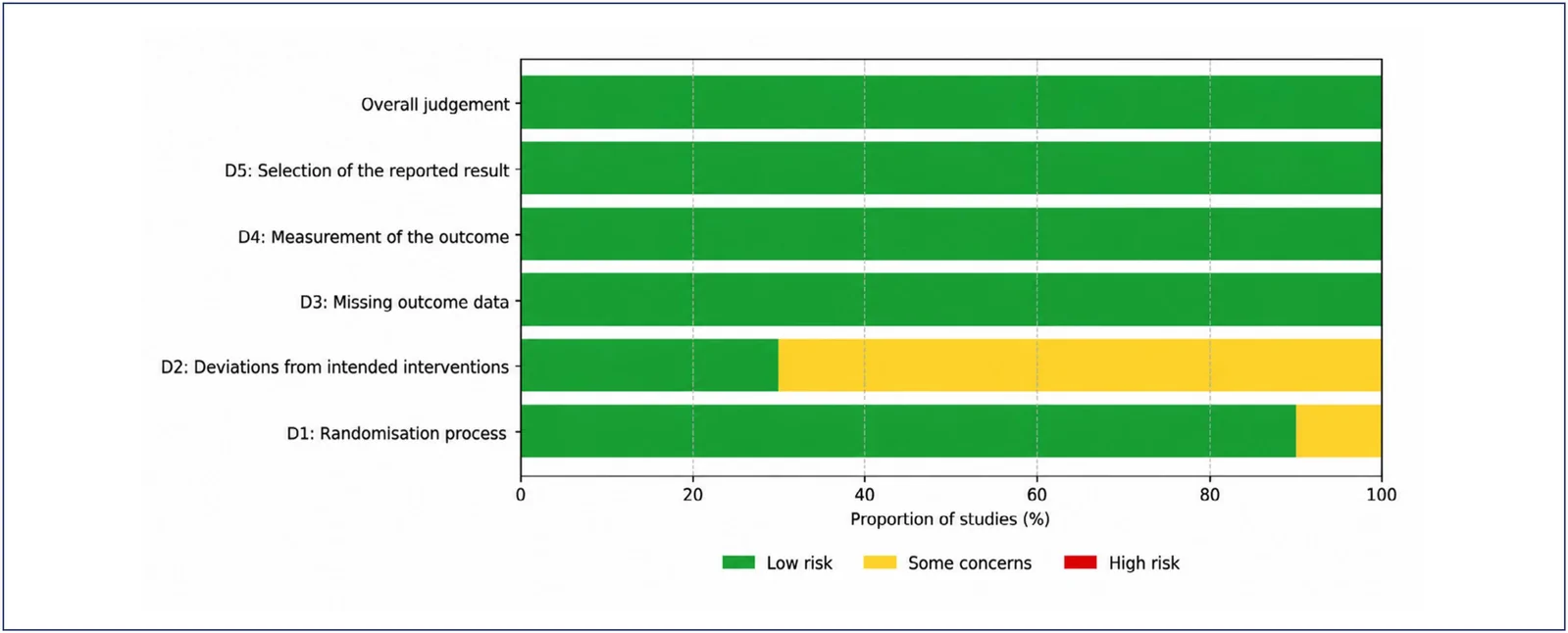
Traffic light plot of risk of bias assessment for each study according to the RoB tool

### Primary outcome – External cephalic version success

Ten randomized controlled trials including 2,722 women (1,359 allocated to adjunct interventions and 1,363 to standard care or control) reported external cephalic version success. In the random-effects meta-analysis, adjunct strategies were associated with a higher probability of successful version (RR 1.53; 95% CI 1.15–2.04; I² = 87%) (Figure 4). According to the GRADE methodology, the certainty of evidence for this outcome was considered low due to serious inconsistency (I² = 87%). The substantial heterogeneity observed across studies likely reflects important clinical and methodological differences. Trials varied with respect to the type of adjunct intervention evaluated, timing of the external cephalic version attempt (early versus term), use and dosage of tocolytic agents, anesthesia protocols, and operator experience. Additionally, variations in study settings and baseline obstetric characteristics may have contributed to differences in effect size. Such diversity is expected in pragmatic obstetric interventions and may partially explain the magnitude of statistical heterogeneity observed.

**Figure 4:**
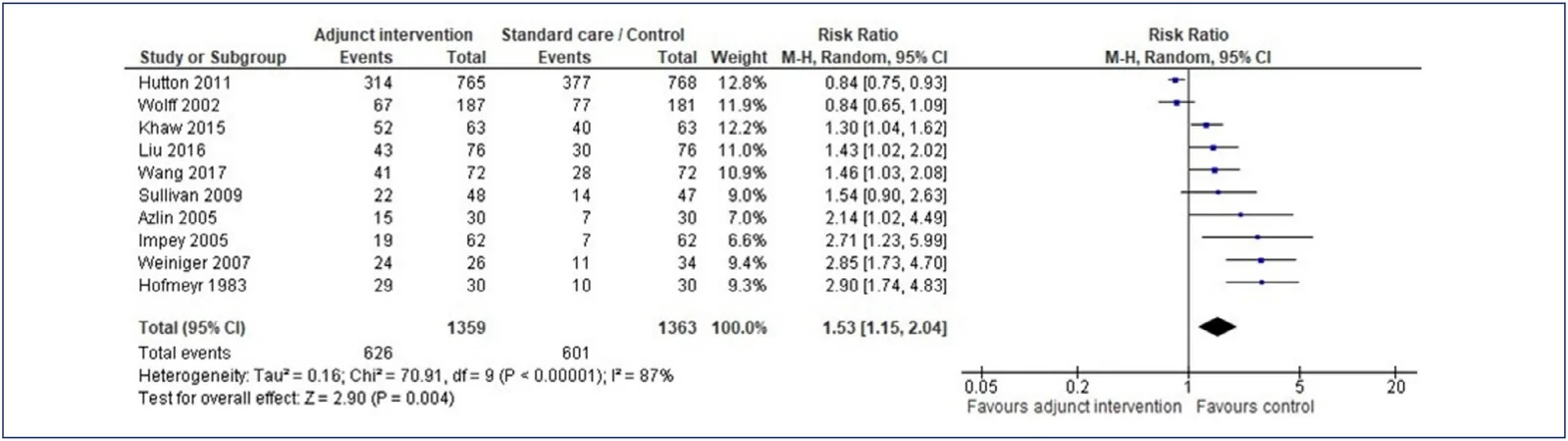
Forest plot of adjunct interventions versus standard care for external cephalic version success (random-effects model). Effect sizes are presented as risk ratios with 95% confidence intervals

### Subgroup analyses

Pharmacological tocolysis was evaluated in two trials (n = 184 participants), with a pooled risk ratio of 2.39 (95% CI 1.39–4.11; I² = 0%). Neuraxial anesthesia was assessed in three trials (n = 217 participants), with a pooled risk ratio of 1.75 (95% CI 1.07–2.85; I² = 73%). Systemic analgesia included three trials (n = 360 participants), with a pooled risk ratio of 1.39 (95% CI 1.15–1.69; I² = 0%). Protocol or non-pharmacological strategies included three trials (n = 1,913 participants), with a pooled risk ratio of 1.18 (95% CI 0.72–1.92; I² = 91%) (Figure 5).

**Figure 5:**
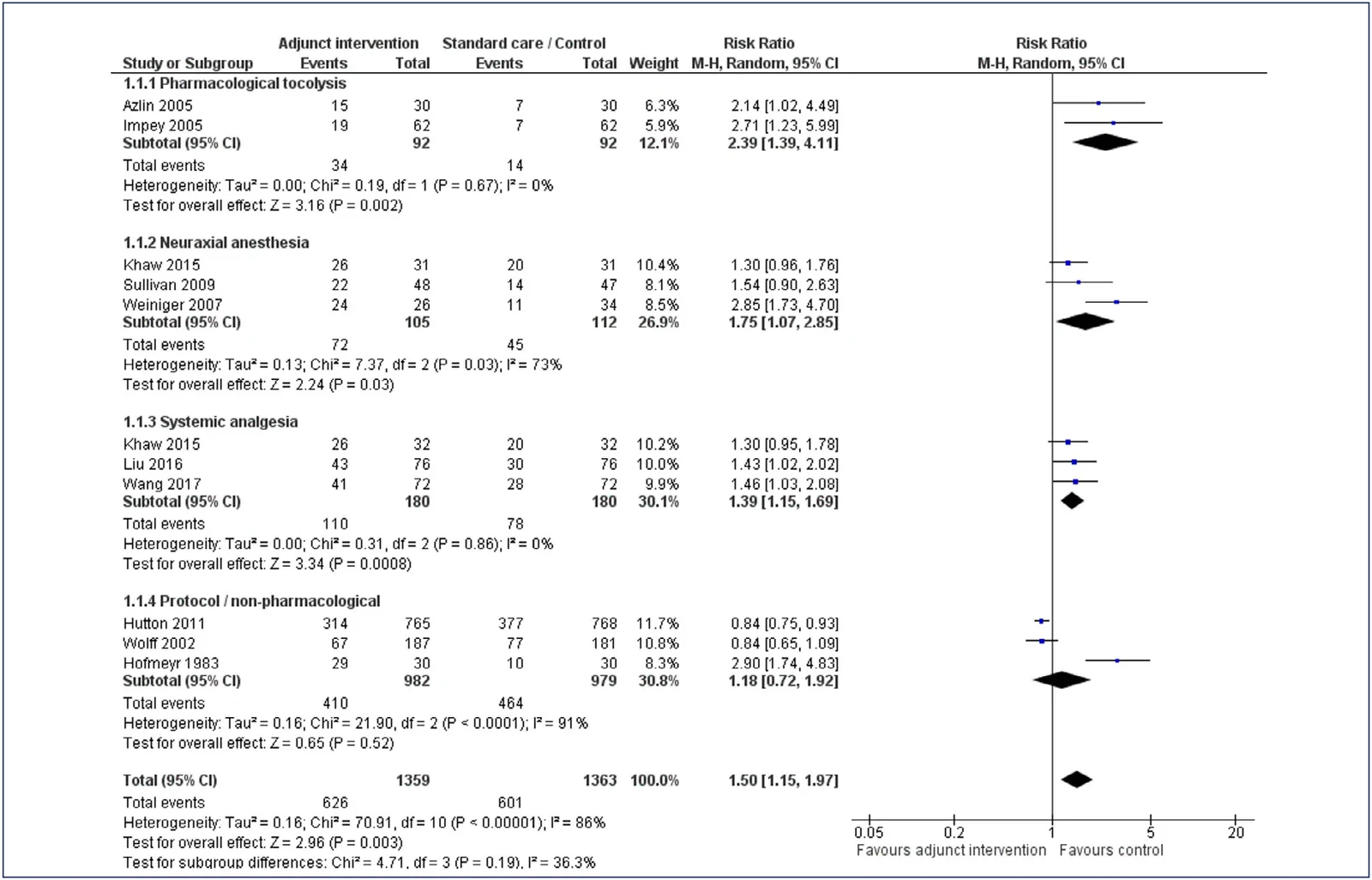
Forest plot of adjunct interventions by intervention category for external cephalic version success (random-effects model). Effect sizes are presented as risk ratios with 95% confidence intervals

### Cesarean delivery

Six randomized controlled trials including 2,240 women reported cesarean delivery. Cesarean delivery occurred in 594/1,121 (53.0%) women receiving adjunct interventions and 641/1,119 (57.3%) controls. The pooled random-effects analysis showed no statistically significant reduction in cesarean risk (RR 0.88; 95% CI 0.76–1.02; I² = 58%) (Figure 6). The certainty of evidence regarding the reduction of cesarean delivery was graded as moderate due to imprecision (95% CI crossing the line of no effect).

**Figure 6:**
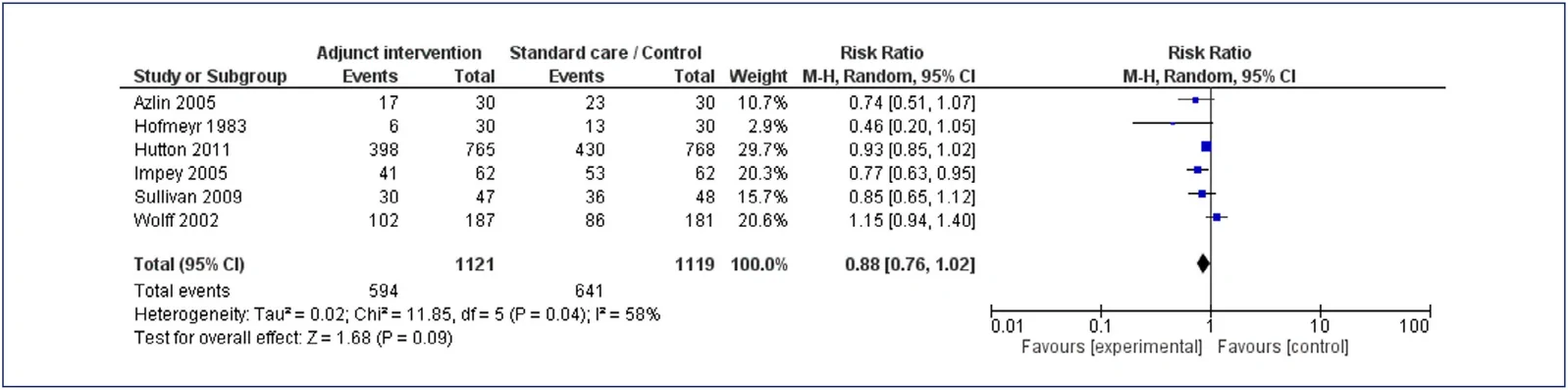
Forest plot of adjunct interventions versus standard care for cesarean delivery (random-effects model). Effect sizes are presented as risk ratios with 95% confidence intervals

### Maternal and neonatal complications

Maternal and neonatal safety outcomes were inconsistently reported across the included trials and were not suitable for quantitative synthesis. The trial by Hutton et al.^(9)^ reported serious fetal complications in 41/765 women in the early ECV group and 31/768 in the delayed ECV group. Serious maternal morbidity occurred in 39/765 and 29/768 women, respectively. No maternal deaths were recorded. Three neonatal deaths were reported, all in the control group. The remaining trials either did not report structured safety outcomes or described only individual adverse events. Studies evaluating systemic analgesia reported events such as nausea, vomiting, dizziness, hypotension, and transient fetal heart rate changes. Consequently, the certainty of evidence for safety outcomes was assessed as low to very low owing to imprecision and sparse data.

## Discussion

External cephalic version (ECV) success depends largely on the clinical conditions under which the maneuver is performed. Our analysis demonstrates that adjunct interventions are consistently associated with higher success rates. The evidence synthesized in this review derives from randomized controlled trials conducted across different settings and employing pharmacological, anesthetic, and protocol-based strategies, and the direction of effect was broadly concordant across studies. Current professional guidance supports the use of adjunctive measures—particularly uterine relaxation—to facilitate version.^(1)^

Among the evaluated approaches, tocolysis produced the most pronounced effect. Beta-adrenergic agonists reduce myometrial contractility and directly decrease resistance to fetal rotation, offering a clear physiological explanation for improved outcomes. Randomized trials and pooled analyses report higher rates of cephalic presentation and fewer cesarean deliveries when beta-stimulants are administered,^(2)^ reinforcing uterine relaxation as a key determinant of procedural effectiveness.

Neuraxial anesthesia also improved success rates. By reducing pain, abdominal wall tone, and maternal guarding, spinal and epidural techniques facilitate operator performance and fetal manipulation. Meta-analyses and randomized trials consistently demonstrate higher success with regional anesthesia compared with no anesthesia,^(3,14,15)^ and these approaches appear particularly useful when combined with tocolysis.^(16)^

The success of ECV is not determined by adjunct interventions alone and, in practice, often depends on baseline obstetric conditions. The procedure tends to become more challenging in situations such as reduced amniotic fluid, anterior placental location, and increased abdominal wall tension—particularly in nulliparous patients—where fetal rotation may be limited despite appropriate technique. In some cases, the maneuver simply does not progress even after repeated, well-executed attempts. More favorable scenarios, including multiparity or a more mobile fetus, usually allow smoother execution, although outcomes remain somewhat unpredictable. These variations are commonly observed in clinical practice and likely contribute to the heterogeneity in success rates reported across studies, reinforcing the importance of careful patient selection when considering ECV.^(17-20)^

Adjustments in timing present additional trade-offs. Earlier attempts increase the likelihood of cephalic presentation at birth but do not consistently reduce cesarean delivery and may increase preterm labor.^(11,21)^ One early randomized trial compared an implementation strategy involving an ECV attempt with expectant management. Although this design did not evaluate a pharmacological or anesthetic adjunct during the maneuver itself, it was considered relevant as a protocol-based approach influencing how ECV is offered in clinical practice. This balance between benefit and risk explains why most recommendations favor performing ECV at term^1^. Importantly, even when technical success improves, reductions in cesarean delivery remain variable, emphasizing that delivery mode depends on several obstetric factors beyond fetal presentation alone.^(22)^

Safety outcomes were generally reassuring. Large cohorts and systematic reviews report low rates of serious complications, including placental abruption, emergency cesarean delivery, and persistent fetal distress, with overall serious adverse events occurring in fewer than 1% of attempts and no increase in perinatal mortality when ECV is performed in appropriate clinical settings.^(17-20)^ These findings support the safety of the procedure when immediate obstetric care is available.

In clinical practice, external cephalic version is generally well tolerated, although its safety profile depends on an adequately prepared clinical environment. Transient fetal heart rate changes are the most common observation; while these usually resolve spontaneously, they require close monitoring during and after the maneuver. Less frequent events such as vaginal bleeding or premature rupture of membranes may occur, and more serious complications—such as placental abruption or the need for emergency delivery—remain rare, typically affecting fewer than 1% of attempts. Even with this low incidence, the possibility of these events supports performing ECV in settings with immediate access to obstetric care and should be considered during patient counseling.^(17-20)^

Methodological quality of the included trials was acceptable overall, which strengthens confidence in the pooled estimates. The heterogeneity observed across analyses likely reflects differences in intervention type, gestational age at attempt, operator expertise, and study protocols. Similar sources of variability have been described in prior systematic reviews of ECV interventions,^(2,16)^ and such diversity should be expected in pragmatic obstetric procedures. Greater standardization of techniques and outcome definitions would facilitate clearer comparisons and more precise estimates of benefit in future research.

ECV outcomes are rarely uniform across different clinical settings. In the real world, results tend to fluctuate based on operator experience, local practices, and the availability of adjunct measures. Some centers consistently report higher success rates, often reflecting not just a higher volume of cases, but greater familiarity with the maneuver and more structured protocols. These differences in patient selection and local routines likely account for much of the heterogeneity observed in our analysis, and they also highlight that results achieved in controlled trials may not always translate directly to all clinical environments.^(19,20)^

Despite the widespread recommendation of ECV as a strategy to reduce cesarean delivery, its implementation remains inconsistent across clinical settings, which may contribute to the gap between expected and observed outcomes.

## Conclusion

Adjunct interventions improve the success of external cephalic version, particularly strategies aimed at promoting uterine relaxation and reducing maternal discomfort. Pharmacological tocolysis and neuraxial anesthesia were consistently associated with higher rates of successful version, whereas the impact on cesarean delivery was less uniform and appeared to depend on multiple obstetric factors beyond fetal presentation alone. Overall, external cephalic version was associated with low rates of serious maternal and neonatal complications when performed in appropriate clinical settings. These findings support the routine consideration of evidence-based adjunct measures to optimize procedural success and reinforce the safety of ECV as a strategy to reduce breech presentation at birth. Further standardization of techniques and outcome reporting may improve comparability across studies and guide future practice.

## Data Availability

The research data are described in the article presented.
