# Supplementary material for: Effectiveness and safety of adjunct interventions for external cephalic version in breech presentation: a systematic review and meta-analysis
Source: Rev Bras Ginecol Obstet. 2026 Oct 20;48:e-rbgo40. doi: 10.61622/rbgo/2026rbgo40 (PMC13690393; doi:10.61622/rbgo/2026rbgo40)
Supplement: Supplementary file 1 [file 1806-9339-rbgo-48-e-rbgo40-Suppl01.pdf]

Supplementary Material

Chart 1S. Detailed electronic search strategies used for each database (MEDLINE via PubMed, Embase, Scopus, Cochrane CENTRAL, and LILACS). The searches were performed from database inception to February 2026 without language or publication date restrictions. Controlled vocabulary terms (MeSH, Emtree, and DeCS) and free-text keywords related to external cephalic version and breech presentation were combined using Boolean operators. Database-specific syntax and field tags were applied as appropriate. Additional filters were not used. The search strategies were designed to maximize sensitivity and ensure comprehensive identification of relevant randomized controlled trials

Database	Search strategy
PubMed (MEDLINE)	(“Version, fetal”[Mesh] OR “external cephalic version”[tiab] OR ECV[tiab]) AND (“Breech Presentation”[Mesh] OR breech[tiab])
Scopus	TITLE-ABS-KEY (“external cephalic version” OR ECV) AND TITLE-ABS-KEY (breech OR “breech presentation”) AND NOT TITLE-ABS-KEY (intrapartum OR “internal version” OR simulation OR mannequin OR training)
Embase	(‘external cephalic version’/exp OR ‘external cephalic version’:ti,ab OR ecv:ti,ab) AND (‘breech presentation’/exp OR breech:ti,ab)
Central	(“external cephalic version” OR ECV) AND (breech OR “breech presentation”)
LILACS	tw:(“external cephalic version” OR ECV OR “versao cefalica externa”) AND tw:(breech OR “breech presentation” OR “apresentacao pelvica” OR “apresentacao podalica”)
